# Vitamin K suppression of ferroptosis in neuronal model systems

**DOI:** 10.1002/alz70855_097616

**Published:** 2025-12-23

**Authors:** Carmen J Narvaez, JoEllen Welsh

**Affiliations:** ^1^ SUNY Albany, RENSSELAER, NY, USA; ^2^ State University of New York at Albany, RENSSELAER, NY, USA

## Abstract

**Background:**

Ferroptosis (iron‐mediated phospholipid peroxidation) has been implicated in neuronal loss associated with Alzheimer's disease (AD). Glutathione Peroxidase‐4 (GPX4) mitigates ferroptosis in healthy neurons, but the activity of this pathway declines with age. Menaquinone‐4 (MK4), a natural form of vitamin K, can be enzymatically reduced by FSP1, VKORC1 and VKORC1L1 to generate the potent ferroptosis suppressor MK4‐H. We hypothesize that vitamin K suppression of ferroptosis becomes critical to neuronal survival as GPX4 and its substrate glutathione decline in aging brain. In these studies we examined whether MK4 acts as a ferroptosis suppressor in murine‐derived HT22 neuronal cells grown as adherent monolayers or as neurospheres.

**Method:**

HT22 cells were acutely exposed to MK4 (≤1µM) in the presence/absence of ferroptosis inducers RSL3 and FIN56 (GPX4 inhibitors), sulfasalazine (xCT inhibitor) and glutamate (NMDAR agonist). Cell density, imaging, flow cytometry and Western blotting were used to assess survival, morphology, ferroptosis‐specific phospholipid peroxidation (Liperfluo) and subcellular expression of FSP1, VKORC1 and VKORC1L1. Sensitivity to RSL3 was also assessed in HT22 cells after differentiation in neurobasal media supplemented with N2, Gln, EGF, bFGF and B27 lacking antioxidants, and when cultured as neurospheres under low attachment conditions.

**Result:**

Ferroptosis was induced in HT22 cells at concentrations of RSL3 as low as 0.01µM (65% reduction in cell density within 20hrs). MK4 (0.05µM) abrogated RSL3 mediated cell loss and phospholipid peroxidation as measured by Liperfluo. Similar protective effects of MK4 were observed in neurospheres and in differentiated HT22 cells. MK4 also protected HT22 cells from sulfasalazine, glutamate and FIN56 which induce ferroptosis via mechanisms distinct from RSL3. GPX4 was detected in both cytosolic (CYT) and non‐nuclear membrane (NNM) fractions of HT22 cells, indicating that it mediates cellular‐wide protection against ferroptosis. FSP1 was strongly expressed in the CYTO fraction whereas VKORC1 and VKORC1L1 were only detected in the NNM fraction.

**Conclusion:**

MK4 (vitamin K2) promotes survival of both proliferating and differentiated HT22 cells via suppression of ferroptosis induced by multiple pathways. GPX4 and the MK4 metabolizing enzymes (FSP1, VKORC1, VKORC1L1) are expressed in HT22 cells and likely cooperate to suppress ferroptosis in distinct cellular compartments.

